# A Self-Adaptive Umbrella Model for Vibration Analysis of Graphene

**DOI:** 10.3390/ma11122497

**Published:** 2018-12-08

**Authors:** Liu Chu, Jiajia Shi, Hang Yu, Eduardo Souza de Cursi

**Affiliations:** 1 School of Transportation, Nantong University, Nantong 226019, China; chuliu@ntu.edu.cn (L.C.); nuaayuhang@163.com (H.Y.); 2 Département Mécanique, Institut National des Sciences Appliquées de Rouen, 76801 Rouen, France; souza@insa-rouen.fr

**Keywords:** self-adaptive umbrella model, vibration analysis, graphene

## Abstract

The beam finite element and molecular dynamics models are two popular methods to represent the reaction of carbon-carbon bonds in graphene. However, the wrinkles and ripples in geometrical characteristics are difficult take into consideration. The out-planar mechanical properties are neglected in classical models of graphene. This paper proposes a self-adaptive umbrella model for vibration analysis of graphene. The parameters in the umbrella model are flexible to adapting the geometrical and material characteristics of graphene. The umbrella model consists of shell and beam elements. The honeycomb beam and planar shell model of graphene are included in the self-adaptive umbrella model as particular cases. The sensitivity analysis and results confirmed the rationality and feasibility of the self-adaptive umbrella model.

## 1. Introduction

Based on its special internal microstructure, graphene has extremely high electron mobility [1,2,3], extraordinary thermal properties [4], and impermeability to gases but optical transparency [5]. Graphene has also been measured to possess significant mechanical properties [6] with two-dimensional (2D) lattice stability [7]. The single layer carbon atoms in graphene are held together by a backbone of overlapping sp^2^ hybrid bonds [8]. Due to the stability of the sp^2^ bonds, the hexagonal lattice is formed with the capacity to resist in-plane deformations [9]. However, the accurate representation of the specific sp^2^ bonds in the microstructure of graphene is a challenge in theoretical investigations.

Based on diverse effective thickness and potential functions of graphene, numerous algorithms and theories have been proposed and developed, such as Quantum Mechanic (QM) calculations, molecular dynamic (MD) simulations, and continuum models (CM). Yanovsky [10] used the QM to study the mechanism of deformation and fracture of graphene. Gao [11] discussed the mechanical properties of monolayer graphene under tensile and compressive loading by QM. In addition, carbon atoms are expressed by tight binding molecular dynamics by Hernandez [12] by MD simulation. Ni [13] reported the anisotropic mechanical properties of graphene sheets from MD simulation. Tsai [14] expressed the mechanical characteristics of graphite using MD simulation. Ansari [15] studied a defective single-layered graphene sheet by using MD simulation. Zhang [16] predicted the mechanical properties of a bilayer graphene sheet coupled by sp^3^ bonding using MD simulation. Javvaji [17] extensively investigated the effect of domain size, lattice orientation, and crack length on the mechanical properties of graphene employing MD simulation. 

Besides QM and MD approaches, CM methods have attracted tremendous attention. For a description of the mechanical properties of graphene sheets, Reddy [18] used Brenner’s potential and Cauchy–Born rule. Natsuki [19] created a continuum model by performing analytical molecular structural mechanics. Arrayo [20] calculated the Young’s modulus and Poisson’s ratio of graphene based on Brenner potential [21] and modified Brenner potential [22]. Chou [23] applied a beam element to represent the relationship between computational chemistry and structural mechanics. Hemmasizadeh [24] developed the equivalent continuum model of a single layer graphene sheet. Georgantzinos [25] performed a numerical investigation of the elastic mechanical properties of graphene structures. Finally, a fully non-linear spring-based finite element prediction for graphene sheets was explored [26].

In terms of the configuration of CM, F. Scarpa proposed truss-type analytical models based on cellular material mechanics theory to describe the in-plane linear elastic properties of the single layer graphene sheets [27]. Meo and Rossi modeled graphene sheets employing translation and torsional spring element to simulate bond stretching and bond angle variations [28]. Rafiee [29] developed a closed-form solution for predicting the Young’s modulus of a graphene sheet in two orthogonal directions by substituting the lattice structure with a honeycomb-like discrete structure. In addition, Pettifor and Oleinik [30] proposed a semi-empirical model based on the tight-binding approach. The Lennard-Jones potential energy has been used to compute the energy between the inter-atomic layers [31].

The analytical results in the literature vary significantly [32] with deviations and fluctuations. It is necessary to develop a comprehensive method with self-adaptive capacity to include uncertainty consideration in the simulation model. On the other hand, the inherent and inevitable crumpling in the out-planar direction of the monolayer is responsible for the deterioration of the mechanical properties of the material [33]. The in-plane beam and plane finite element model are limited due to neglect of the geometrical complexity. Therefore, to solve these problems, a self-adaptive umbrella model with hybrid finite elements is proposed for vibration analysis of graphene.

In this paper, the formation of the self-adaptive umbrella model is introduced. The model configurations are described by hybrid finite elements with corresponding parameters. The self-adaptive umbrella model extended the classical honeycomb beam finite element model to a three-dimensional out-planar model. The classical honeycomb beam finite element and planar shell element model are included in the self-adaptive umbrella model as special cases. Based on a Monte Carlo simulation, the uncertainties in parameters related with geometrical and material properties are taken into consideration. The sensitivity analysis not only selected the essential input variables in the self-adaptive umbrella model, but also proved the efficacy of the proposed model. The vibration modes of three particular cases in the self-adaptive umbrella model are compared and discussed.

## 2. Model Formation

### 2.1. Model Configuration

**Figure 1:**
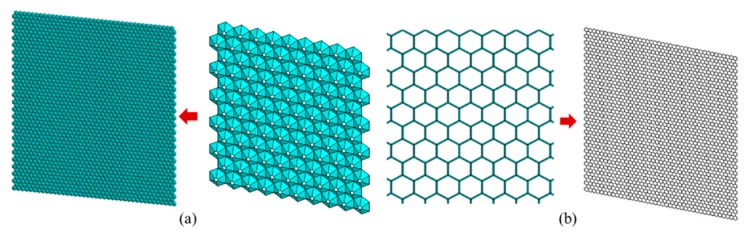
Graphene lattice ((**a**) shows umbrella cells; (**b**) shows beam elements).

**Figure 2:**
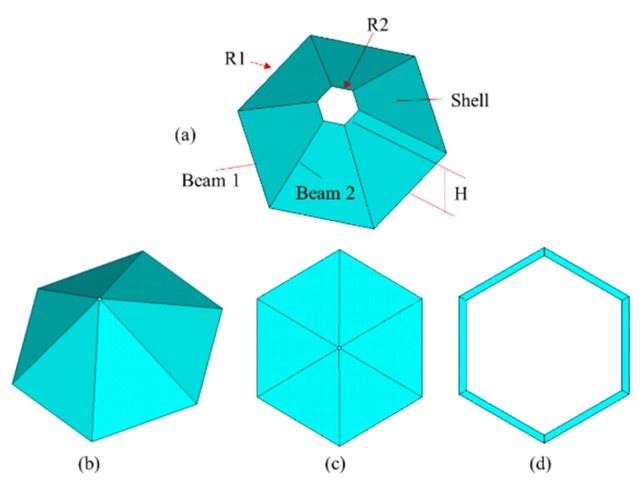
Configuration of graphene microstructure cell ((**a**) is a normal cell, (**b**) is for $R_{2}\to 0$, (**c**) is for $R_{2}\to 0\;(H\to 0$, (**d**) is for $R_{2}\to R_{1}\;,\;H\to 0$).

In the general honeycomb lattice structure of graphene, the carbon-carbon (C-C) bonds are presented by beam elements, as shown in Figure 1b. In order to create the self-adaptive umbrella model for graphene, more complicated configurations are depicted in Figure 1a. The self-adaptive umbrella model consists of more parameters corresponding to geometrical and material properties. The hybrid finite elements (Beam 1, Beam 2, and Shell) are included in the umbrella model. The beam finite element model and the planar shell element model are the special cases of umbrella model as represented in Figure 2.

As presented in Figure 2a, the normal self-adaptive umbrella model for graphene consists of the Beam 1, Beam 2, and Shell finite elements. Besides the parameters corresponding to material properties, such as Young’s modulus, Poisson ratio, and physical density, there are parameters related to the geometrical properties. *R_1_* and *R_2_* are the radii of hexagons in the bottom and top, respectively. According to the geometrical characteristics of hexagons, the radii of hexagons are equal to that of the corresponding length of each side, as demonstrated in Figure 2. The configuration of the umbrella model can be reformed by the change of the geometrical parameters. For example, when the radius of the top in the umbrella model approaches zero, the shape of the umbrella model is expressed as Figure 2b. In addition, Figure 2c,d are unique to the umbrella model. The three-dimensional umbrella model can degrade to a two-dimensional planar model, as shown in Figure 2c, or the beam hexagon model, as shown in Figure 2d. Therefore, the umbrella model proposed in this study has self-adaptive capabilities, and includes the two-dimensional shell and beam model as special cases.

The self-adaptive capacity in the proposed umbrella model for graphene not only contains the classic honeycomb beam finite element models and other in-plane models as special cases, but also has flexibility, in terms of material properties, to explore the relationships between parameters and different elements in the model. The three dimensional geometrical configurations (including two dimensional models as special cases) in the umbrella model are described with the introduced parameters, and the relationships between parameters corresponding to geometrical and material properties are discussed in the sensitivity analysis. The self-adaptive umbrella model is a flexible and feasible method to find appropriate descriptions for the exact microstructure of graphene. 

### 2.2. Model Parameters

**Figure 3:**
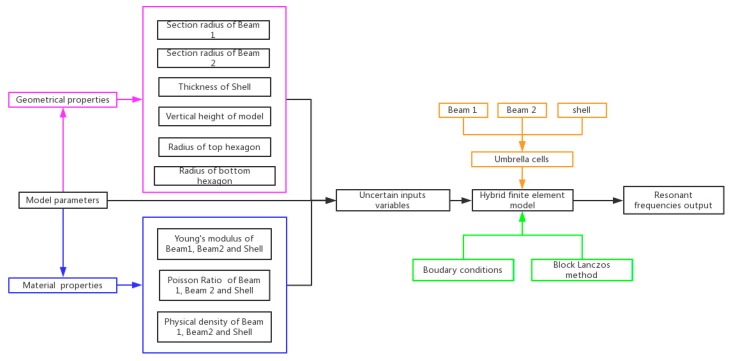
Parameters in the self-adaptive umbrella model for graphene.

In order to precisely describe the self-adaptive umbrella model, parameters are listed in Figure 3. The total parameters are divided into two groups: the geometrical and material parameters. In the first group, there are sectional radii of Beam 1 and Beam 2, thickness of shell element, and the vertical height of model. The radii of the top and bottom hexagons are important parameters to describe the geometrical characteristics of the umbrella model. In the other group, the Young’s modulus, Poisson ratio, and physical density of Beam 1, Beam 2, and Shell, respectively, are the parameters related with material properties of the umbrella model. 

The above parameters are uncertain input variables with certain interval ranges. The umbrella hybrid finite element model is meshed by Beam 1, Beam 2, and shell finite elements. By the Block Lanczos method [34], the resonant frequencies of graphene can be captured as the output results. The Block Lanczos method is feasible to solve the eigenvalue problems in the free vibration of structure. The boundary conditions of the umbrella model are the same as in the classical honeycomb beam finite element model, and the freedom in the four sides of the rectangular graphene sheets is constrained as zero. The interval ranges of each parameter are provided in Table 1. Among the parameters, the diameter of bottom hexagon, 2*R_1_*, is settled as 0.27 nm, to be in agreement with the relevant literature [22,35,36]. *RR* is introduced to represent the ratio between the radius of top and bottom hexagons. In addition, the sectional radius of Beam 1 and Beam 2 is set at half of the thickness of the Shell to satisfy the geometric compatibility. The interval range of the thickness of Shell is 0.001~0.04 nm with the assumption of a thin shell. However, the vertical height of the model fluctuates in a large interval range. In the three-dimensional umbrella model, more emphasis is put on the vertical height of the model. The intervals of material parameters are set according to the experimental data [6]. The wide intervals for the material and geometrical parameters are provided to make sure the exact values of the corresponding parameters are included. The output parameters are the first to fourth resonant frequencies (RF).

The Monte Carlo simulation is rooted from the integrals computation by random number generation.

(1) $$F=\int_{a}^{b}h(x)dx$$

In the interval range (a, b), if h(x) is decomposed into the production of a function f(x) and a probability density distribution function p(x), the above integral can be expressed as,

(2) $$F=\int_{a}^{b}h(x)dx=\int_{a}^{b}f(x)p(x)dx=E_{P(x)}\left[f(x)\right]$$

A large number of samples for the variables from the probability density p(x) are provided, then

(3) $$F=\int_{a}^{b}h(x)dx=E_{P(x)}\left[f(x)\right]≃\frac{1}{n}\sum_{i=1}^{n}f(x_{i})$$

The integral I(y)=∫f(y|x)p(x)dx can be approximated by Monte Carlo simulation as,

(4) $$\hat{I}(y)=\frac{1}{n}\sum_{i=1}^{n}f(y|x_{i})$$

The estimated Monte Carlo standard error can be expressed as

(5) $$\varepsilon^{2}=\frac{1}{n}\left(\frac{1}{n-1}\sum_{i=1}^{n}\left(f(y|x_{i}\right)-\hat{I}(y){)}^{2}\right)$$

If the number of sample (n) is sufficiently large, the Monte Carlo standard error is tiny. Based on the interval ranges of each parameter, a Monte Carlo simulation is used to extract samples for the umbrella model according to the uniform distribution. The size of samples for each input variable in the umbrella model is 1000 to create a reliable sampling space with a confidence interval of 95%. The uniform distribution of input variables in the interval ranges avoids the loss of representative and important values of geometrical and material parameters.

### 2.3. Mathematical Description

**Figure 4:**
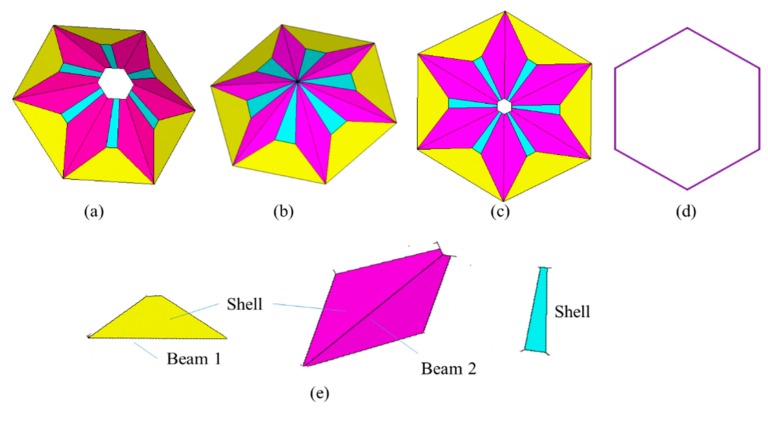
Hybrid finite element model ((**a**) is a normal cell, where (**b**) is for $R_{2}\to 0$, (**c**) is for $R_{2}\to 0,\;H\to 0$, (**d**) is for $R_{2}\to R_{1},\;H\to 0$), (**e**) is for hybrid finite elements in the umbrella model. Yellow denotes the hybrid element with Beam 1 and Shell finite elements; pink presents the denotes element with Beam 2 and Shell finite elements; and blue color represents the independent shell finite element).

The umbrella cells in the self-adaptive model for graphene are meshed by hybrid finite elements, as shown in Figure 4. The yellow components are meshed by the Beam 1 and shell elements; the pink hybrid finite elements combine Beam 2 with shell finite elements, while the blue parts represent the shell finite element independently. By tracking the hybrid finite element model in the self-adaptive umbrella model for graphene, the sensitivity of each finite element to the resonant frequencies is observed, and a more appropriate and precise model can be determined for the modal analysis of graphene. Furthermore, Figure 4b–d express special situations in the self-adaptive umbrella model with specific geometrical parameters.

Based on principal of virtual work, the weak form of the govern equation can be written as [37]

(6) $$\int_{0}^{L}EI\frac{\partial \theta }{\partial x}\delta (\frac{\partial \theta }{\partial x})dx+\int_{0}^{L}\kappa GA(\frac{\partial \xi }{\partial x}-\theta )\delta (\frac{\partial \xi }{\partial x}-\theta )dx=\int_{0}^{L}\delta \xi \rho A\ddot{\xi }dx+\int_{0}^{L}\delta \theta \rho I\ddot{\theta }dx$$

where ξ and θ are the transversal displacement and transversal rotation, while $\ddot{\xi }$ and $\ddot{\theta }$ are the transverse and rotary accelerations, respectively. L is the length of the beam, and δ denotes that the terms are virtual. *E*, I and *G* are the Young’s modulus, the inertia moment, and the shear modulus, respectively, while ρ is the density, A is the cross section area, and κ is the shear coefficient.

For free vibration,

(7) $$Ku-M\ddot{u}=0$$

where K, M are the global stiffness and mass matrices.

The normal solution can be written as

(8) $$u=\phi_{k}e^{iw_{k}}$$

Then,

(9) $$[K-w_{k}^{2}M]\phi_{k}=0$$

where $\phi_{k}$ is a set of displacement-type amplitude at the control points, and $w_{k}$ is the resonant frequency associated with the *k*th mode. Solving the above equation is an eigenvalue problem:

(10) $$\left|K-w_{k}^{2}M\right|=0$$

The discretization of the governing equation can be accomplished by finite elements. For the beam finite element, there are six degrees of freedom for each node. The approximated solution for transversal displacement and rotation can be represented as

(11) $$\left\{ \begin{array}{l} \xi =\sum_{i=1}\varphi_{i}\xi_{i}\\ \theta =\sum_{i=1}\varphi_{i}\theta_{i} \end{array}\right.$$

Consider the natural coordinate ς=[-1, 1] in the element domain:

(12) $$\begin{array}{l} \xi (\varsigma )=\varphi_{1}(\varsigma )\xi_{1}+\varphi_{2}(\varsigma )\xi_{2}\\ \theta (\varsigma )=\varphi_{1}(\varsigma )\theta_{1}+\varphi_{2}(\varsigma )\theta_{2} \end{array}$$

It can be expressed in the matrix form:

(13) $$\left\{\begin{array}{l} \xi (\varsigma )\\ \theta (\varsigma ) \end{array}\right\}=[H]\left\{u\right\}$$

where [H] is the matrix of shape functions and {u} is vector of nodal displacements.

(14) $$[H]=\left[\begin{array}{cc} \begin{array}{ccc} \varphi_{1} & 0 & \varphi_{2} \end{array} & 0\\ \begin{array}{ccc} 0 & \varphi_{1} & 0 \end{array} & \varphi_{2} \end{array}\right]\;\;,\left\{u\right\}=\left\{\begin{array}{c} \xi_{1}\\ \theta_{1}\\ \xi_{2}\\ \theta_{2} \end{array}\right\}$$

By substituting to the weak form of equation, the stiffness and mass matrix in element domain are written as

(15) $$\begin{array}{l} {}[K^{e}]\;=\int_{-1}^{1}{\left[B\right]}^{T}\left[D\right]\left[B\right]\left|J\right|d\varsigma \\ {}[M^{e}]\;=\int_{-1}^{1}\rho I{\left[H\right]}^{T}\left[H\right]\left|J\right|d\varsigma +\int_{-1}^{1}\rho A{\left[H\right]}^{T}\left[H\right]\left|J\right|d\varsigma \end{array}$$

where |J| is the Jacobian determinant, the strain displacement matrix [B] and constitutive matrix [D] are computed from

(16) $$\begin{array}{l} \left[B\right]=\left[\begin{array}{cc} \begin{array}{ccc} 0 & \frac{1}{\left|J\right|}\left(\frac{d\varphi_{1}}{d\varsigma }\right) & 0 \end{array} & \frac{1}{\left|J\right|}\left(\frac{d\varphi_{2}}{d\varsigma }\right)\\ \begin{array}{ccc} \frac{1}{\left|J\right|}\left(\frac{d\varphi_{1}}{d\varsigma }\right) & -\varphi_{1} & \frac{1}{\left|J\right|}\left(\frac{d\varphi_{2}}{d\varsigma }\right) \end{array} & -\varphi_{2} \end{array}\right]\\ \left[D\right]=\left[\begin{array}{cc} EI & 0\\ 0 & \kappa GA \end{array}\right] \end{array}$$

Besides the beam finite element (Beam 1 and Beam 2), there are shell elements (special plane element) in the umbrella model. The strain and displacement relation can be obtained from

(17) $$\varepsilon =\left\{\begin{array}{c} \varepsilon_{p}\\ 0 \end{array}\right\}+\left\{\begin{array}{c} z\varepsilon_{b}\\ \gamma \end{array}\right\}$$

(18) $$\varepsilon_{p}=\left\{\begin{array}{c} u_{0,x}\\ v_{0,y}\\ u_{0,y}+v_{0,x} \end{array}\right\}\;,\;\varepsilon_{b}=\left\{\begin{array}{c} \beta_{x,x}\\ \beta_{y,y}\\ \beta_{x,y}+\beta_{y,x} \end{array}\right\}\;,\;\gamma =\left\{\begin{array}{c} \beta_{x}+w_{0,x}\\ \beta_{y}+w_{0,y} \end{array}\right\}$$

where u,v,w are the displacements components in the x,y,z axes. β is the transversal normal rotation. For the free vibration, the weak form for plane and shell is written as

(19) $$\int_{\Omega }\delta \varepsilon^{T}D\varepsilon d\Omega +\int_{\Omega }\delta \gamma^{T}D^{s}\gamma d\Omega =\int_{\Omega }\delta u^{Τ}m\ddot{u}d\Omega$$

where

(20) $$\varepsilon =\left[\begin{array}{c} \varepsilon_{p}\\ \varepsilon_{b} \end{array}\right]\;\;,\;D=\left[\begin{array}{cc} D^{m} & \bar{B}\\ \bar{B} & D^{b} \end{array}\right],\;D^{s}=\int_{-h/2}^{h/2}D_{s}(z)dz$$

With

(21) $$D_{m}(z)=\frac{E}{1-v^{2}}\left[\begin{array}{ccc} 1 & v & 0\\ v & 1 & 0\\ 0 & 0 & \left(1-v\right)/2 \end{array}\right],\;D_{s}(z)=\frac{kE}{2(1+v)}\left[\begin{array}{cc} 1 & 0\\ 0 & 1 \end{array}\right]$$

(22) $$\begin{array}{l} D^{m}=\int_{-h/2}^{h/2}D_{m}(z)dz,\;\bar{B}=\int_{-h/2}^{h/2}zD_{m}(z)dz(z),\\ D^{b}=\int_{-h/2}^{h/2}z^{2}D_{m}(z)dz \end{array}$$

Based on the finite elements, the resonant frequencies of graphene are computed in the umbrella model. The parameters in the self-adaptive umbrella model are supposed to be independent. Because the number of introduced parameters in the umbrella model is larger than the number of provided equations, the analytical solutions for each parameter are difficult to obtain. Therefore, specific interval ranges for parameters are used to detect the sensitivity and importance of each parameter to the resonant frequencies in the vibration of graphene. 

## 3. Results and Discussion

### 3.1. Sensitivity Analysis

The number of the input variables in the self-adaptive umbrella model for graphene is 12. The sensitivity levels of each input variable to the resonant frequencies of graphene are different; therefore, it is necessary to distinguish the importance of the input variables. Based on the database of the Monte Carlo simulation of the self-adaptive umbrella model, the first order regression fitting coefficients of independent input variables are obtained from Equation (23), and are listed in Table 2.

(23) $$F_{i}=a_{ij}X_{j}\;(i=1,\cdots ,4;\;j=1,\cdots ,12)$$

(24) $$F_{i}=\sum_{j=1}^{12}b_{ij}X_{j}\;(i=1,\cdots ,4)$$

In Table 2, the Young’s modulus of Beam 1 and Shell, Poisson ratio of Beam 1, Beam 2, and Shell, vertical height of model and sectional radius of Beam 1 have positive effects on the resonant frequencies of graphene. In contrast, the Young’s modulus of Beam 2, physical density of Beam 1, Beam 2, and Shell and the ratio between the radius of top and bottom hexagons have negative effects on the resonant frequencies of graphene. 

With the increase of the Young’s modulus of Beam 1 and Shell (*E_1_*, *E_3_*), Poisson ratio of Beam 1, Beam 2 and Shell (*V_1_*, *V_2_*, *V_3_*), vertical height of model (*H*), and sectional radius of Beam 1 (*T_1_*), the first to the fourth resonant frequencies are all amplified. Among them,* E_1_*, *V_2_*, *H* and *T_1_* are the more important parameters with larger fitting coefficients in the self-adaptive umbrella model for graphene. With the enlargement of the Young’s modulus of Beam 2 (*E_2_*), physical density of Beam 1, Beam 2 and Shell (*P_1_*, *P_2_*, *P_3_*), and the ratio between the radius of top and bottom hexagons (*RR*) cause the reduction of the first to the fourth resonant frequencies in graphene. Furthermore, *P_1_* and RR have more significant effects on the resonant frequencies than *E_2_*, *P_2_*, and *P_3_*.

The above results of the sensitivity analysis are based on the first order fitting between the independent parameters with the resonant frequencies in the Monte Carlo simulation sampling space. However, the relationship between the input variables with the resonant frequencies in graphene is more complicated; there are correlations between parameters. Therefore, the global regression including all parameters is used, as shown in Equation (24). Table 3 lists the regression coefficients of input variables in the self-adaptive umbrella model.

In order to be clearer, parameters are divided into two groups according to the regression coefficients in Table 3. In first group, the vertical height of model (*H*), Young’s modulus of Beam 1 (*E_1_*), section radius of Beam 1 (*T_1_*), and Poisson ratio of Beam 2 (*V_2_*) play positive roles in resonant frequencies, while in the second group, the physical density of Beam 1 (*P_1_*), the ratio between the radius of top and bottom hexagons (RR), and Poisson ratio of Shell (*V_3_*) have negative effects on the resonant frequencies of graphene. Moreover, the remaining parameters, namely Poisson ratio of Beam 1 (*V_1_*), Young’s modulus of Beam 2 and Shell (*E_2_*, *E_3_*) and physical density of Beam 2 and Shell (*P_2_*, *P_3_*), may be neglected.

The results of independent parameter fitting are compared with those of global regression. There is a good agreement among some parameters in the first to fourth resonant of graphene. The coefficients of Young’s modulus of Beam 1 and Beam 2 (*E_1_*, *E_2_*), Poisson ratio of Beam 1 (*V_1_*), section radius of Beam 1 (*T_1_*), and physical density of Beam 1, Beam 2, and Shell (*P_1_*, *P_2_*, *P_3_*) are close in independent parameter fitting and global regression. However, discrepancies appear in the other parameters, especially in the vertical height of model (*H*), and the Poisson ratio of Beam 2 and Shell (*V_2_*, *V_3_*). However, there is no doubt that the Young’s modulus of Beam 1 (*E_1_*), vertical height of model (H), Poisson ratio of Beam 2 (*V_2_*), section radius of Beam 1 (*T_1_*), the ratio between the radius of top and bottom hexagons (*RR*), and the physical density of Beam 1 (*P_1_*) have larger absolute values in fitting coefficients than other parameters, and play more important roles in the resonant frequencies of graphene. 

**Figure 5:**
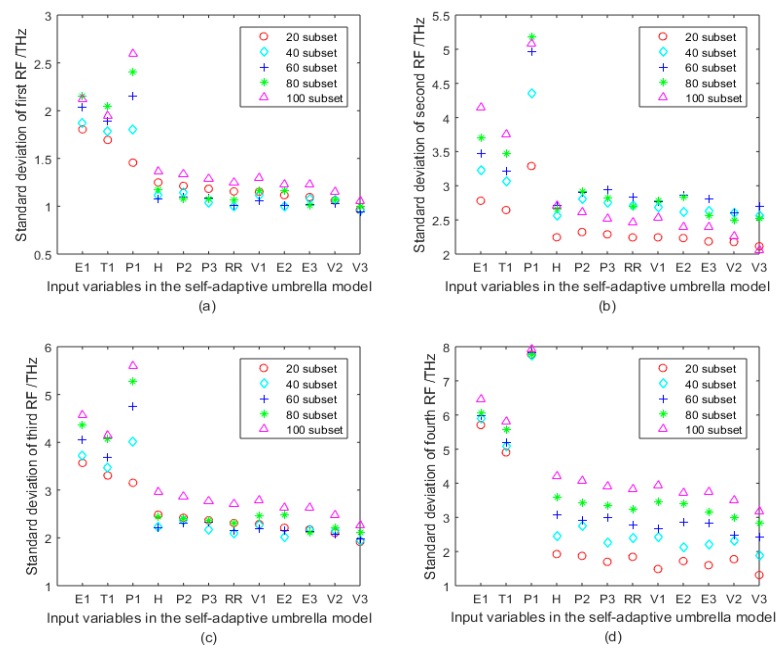
Variance of resonant frequencies in subsets of Monte Carlo simulation. (**a**–**d** are for the standard deviation of the first, second, third and fourth RF, respectively).

The variances of resonant frequencies in subsets of the Monte Carlo-based finite element model are illustrated in Figure 5. The subsets of resonant frequencies are selected according to the corresponding parameters. The variances of resonant frequencies in subsets are important indexes to evaluate the parameter sensitivity. With the amplification of the number of subsets, the variances of the first to fourth resonant frequencies of graphene have different degrees of increment. The results of variances of resonant frequencies demonstrate that the Young’s modulus of Beam 1 (*E_1_*), section radius of Beam 1 (*T_1_*), the physical density of Beam 1 (*P_1_*), and the vertical height of model (H) are more sensitive to impacts on the resonant frequencies of graphene, which are consistent with the above results. 

In a word, the parameters corresponding with Beam 1 (*E_1_*, *T_1_*, *P_1_*) except Poisson ratio are critical to the self-adaptive umbrella model for graphene. On the one hand, the self-adaptive umbrella model for graphene is compatible with the classical honeycomb beam finite element model. The honeycomb beam finite element model is an effective model to represent the important characteristics of mechanical properties in graphene. As an advanced method, the self-adaptive umbrella model for graphene includes the honeycomb beam finite element model as a special case. On the other hand, the vertical height of model (*H*) in the self-adaptive umbrella model also plays an essential role in resonant frequencies of graphene. The vertical height of model (*H*) describes the geometrical characteristics of ripples and wrinkles in graphene. Therefore, the self-adaptive umbrella model for graphene is a smart method, with consideration of ripples or wrinkles as well, as the function of honeycomb beam finite element model.

### 3.2. Result Discussion

**Figure 6:**
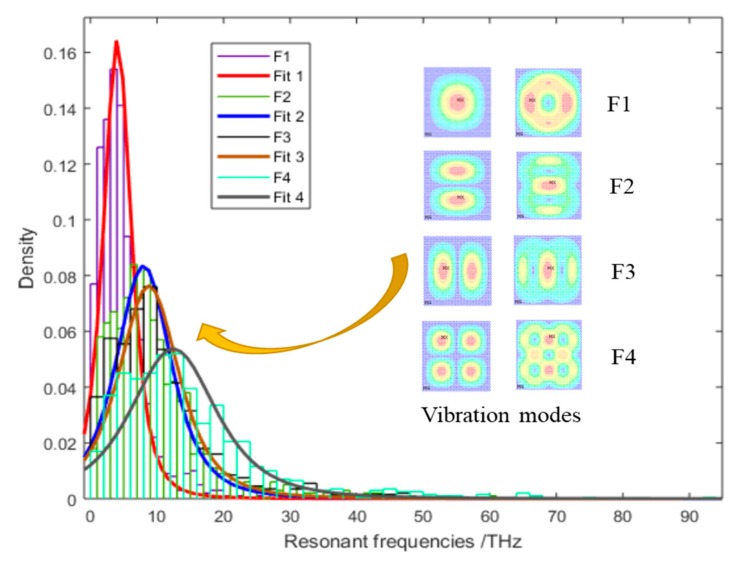
Probability results of resonant frequencies in Monte Carlo simulation.

Based on Monte Carlo simulation, the probability density distribution results of resonant frequencies in graphene are illustrated in Figure 6. The interval ranges of resonant frequencies for probability density distribution become wide from the first to the fourth order vibrations. The probability density distribution of resonant frequencies are more concentrated in the left small value range, but with a long drag in the right large value range. This means that the self-adaptive umbrella model has a strong probability of showing the results of resonant frequencies limited in 0–10 THz, and has a weak probability of having resonant frequencies in wide interval ranges beyond 10 THz. The scope of resonant frequencies computed by the self-adaptive umbrella model confirms its appropriateness and feasibility for vibration analysis of graphene.

The statistic results of the Monte Carlos simulation in the self-adaptive umbrella model for graphene are compared with the reported results in literatures [37,38,39,40,41,42,43] in Table 4. Among them, Wei [37], Liu [40] and Kudin [41] applied the density functional theory; Gupta [38] and Khattibi [39] used the molecular dynamics; and molecular mechanics are performed by Reddy [18], Cadelano [42] and Chu [43]. The maximum, minimum, mean values, and variances of resonant frequencies of the umbrella model in the Monte Carlo simulation are provided. The reported resonant frequencies of graphene in the published literature fall in the interval range of the self-adaptive umbrella model. Thus, the comparability of the self-adaptive umbrella model with models in the reported literature is attested. The umbrella model proposed in this study is shown to be capable of predicting the resonant frequencies of graphene. However, the minimum, maximum, and even mean values of the resonant frequencies for graphene are far from the reported literatures because of the variable dispersion in the certain interval. Further exploration in the determination of the exact value for the parameters in the self-adaptive umbrella model will be performed in subsequent studies.

In order to observe the vibration modes of the self-adaptive umbrella model for graphene, three special cases are computed by performing finite element simulation. In Case 1, the vertical height of the model (*H*) equals to 0.2 nm and the ratio between the bottom and top hexagons (*RR*) is set as 0.3. Case 1 is a normal self-adaptive umbrella model. In Case 2, the vertical height of the model (*H*) stays as 0.2 nm, but the ratio between the bottom and top hexagons (*RR*) is supposed to be 0.8. Case 2 is closer to the classical honeycomb beam finite element model. In Case 3, the vertical height of the model (*H*) changes to 0.001 nm. The ratio between the bottom and top hexagons (*RR*) is set as 0.02. Case 3 is an approximated plane model. The remaining parameters corresponding to the material and geometrical properties are equal in the three cases. These three cases are typical situations in the self-adaptive umbrella model.

**Figure 7:**
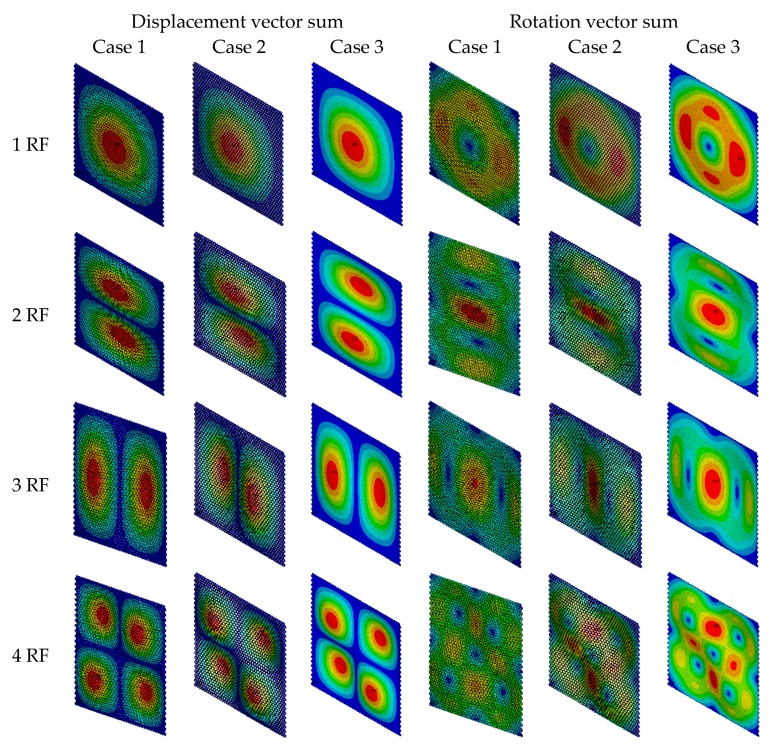
Contour results of self-adaptive umbrella model for graphene.

**Figure 8:**
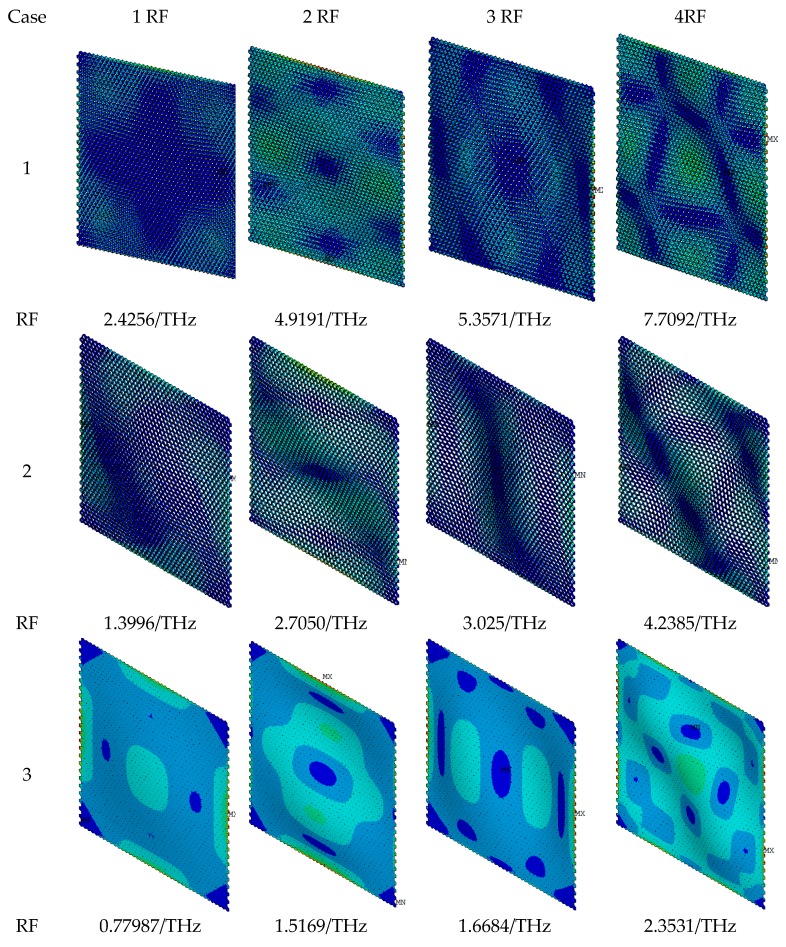
Von Mises stress of self-adaptive umbrella model for graphene.

The displacement vector sums and the rotation vector sums for the first four vibration modes are presented in Figure 7. The differences in displacement vector sums for vibration modes in three cases are not evident. However, the rotation vector sums in three cases are distinct. Figure 8 exhibits the Von Mises stress and resonant frequencies of the self-adaptive umbrella model for graphene. In the same order of vibration modes, the resonant frequencies in Case 1 are larger than those in Case 2 and Case 3. Moreover, the results of Von Mises stress in the vibration modes demonstrate the wrinkles in graphene. 

Furthermore, by comparing the resonant frequencies of the umbrella model for graphene in the three special cases with those in the literatures, the results of Case 2 are closer to the results of density functional theory, molecular dynamics, and molecular mechanics, especially the results provided by Reddy [18], Cadelano [42] and Kudin [41], while in Case 2, the vertical height of the umbrella model for graphene is 0.2 nm. As mentioned in the model formation, if the vertical height of the umbrella model is close to zero, the umbrella model is degraded into the two-dimensional model. In contrast, when the vertical height of the umbrella model is not zero, the three-dimensional configuration of graphene is well described by the geometrical parameters in the proposed umbrella model. The results of resonant frequencies prove that the three-dimensional umbrella model is appropriate for vibration analyses of graphene. The increment of computational cost in the umbrella model compared to that of the classical beam finite element is not evident. Therefore, the umbrella model with hybrid finite elements is feasible and appropriate to describe the three-dimensional geometrical characteristics of graphene. 

## 4. Conclusions

In this paper, a self-adaptive umbrella model is proposed for vibration analysis of graphene. The classical honeycomb beam finite element model is extended into a three-dimensional out-planar model with functions to describe the wrinkles or ripples in graphene. The hybrid finite elements with corresponding parameters are applied to construct more appropriate models. The parameters related to the geometrical and material properties are discussed in the sensitivity analysis. The self-adaptive umbrella model is confirmed to have advantages in three aspects. First, the proposed model has the ability to describe the out-planar geometrical characteristics in graphene. Second, satisfactory compatibility to the classical honeycomb beam finite element and shell planar model is attested to. Third, the results of resonant frequencies in graphene are located in a reasonable interval range even, though the input variables are distributed over a wide range. Therefore, the self-adaptive umbrella model is a feasible and appropriate model for mechanical analysis of graphene. 

## Figures and Tables

**Table 1:** Interval ranges of input variables in the self-adaptive umbrella model for graphene.

Definition	Expression	Interval Range	Units
Section diameter of Beam 1	*T_1_*	0.001~0.04	nm
Section diameter of Beam 2	*T_2_*	0.001~0.04	nm
Thickness of Shell	*T_3_*	0.001~0.04	nm
Vertical height of model	*H*	0.001~1.5*R_1_*	nm
Radius of top hexagon	*R_2_*	0.05*R_1_*~0.8*R_1_*	nm
Young’s modulus of Beam 1, Beam 2 and Shell	*E_1_*, *E_2_*, *E_3_*	1000~1.5 × 10^12^	Pa
Poisson ratio of Beam 1, Beam 2 and Shell	*V_1_*, *V_2_*, *V_3_*	0.1–0.4	/
Physical density of Beam 1, Beam 2 and Shell	*P_1_*, *P_2_*, *P_3_*	100–4000	Kg/m^3^

**Table 2:** First order fitting coefficients of input variables in the self-adaptive umbrella model.

	*F_1_*	*F_2_*	*F_3_*	*F_4_*
E_1_* × 10^12^/TPa	3.962	7.787	8.559	12.12
E_2_* × 10^12^/TPa	−0.164	−0.3191	−0.3406	−0.4842
E_3_* × 10^12^/TPa	0.2622	0.5229	0.5705	0.8151
*V_1_*	0.1994	0.4361	0.4946	0.7278
*V_2_*	3.331	6.463	7.185	10.11
*V_3_*	0.7192	1.409	1.534	2.136
P_1_* × 10^3^/TPa	−1.778	−3.484	−3.837	−5.427
P_2_* × 10^3^/TPa	−0.2167	−0.4286	−0.4631	−0.6604
P_3_* × 10^3^/TPa	−0.2171	−0.4253	−0.4681	−0.6613
*H/*nm	3.831	8.372	8.914	13.31
*RR*	−0.5665	−1.483	−1.340	−2.325
T_1_* × 10^−2^/nm	1.379	2.630	2.893	4.035

**Table 3:** Regression coefficients of input variables in the self-adaptive umbrella model.

	*F_1_*	*F_2_*	*F_3_*	*F_4_*
*Z_0_*	2.6054	5.2502	5.6752	8.2169
E_1_* × 10^12^/TPa	3.8416	7.5431	8.2937	11.7405
E_2_* × 10^12^/TPa	−0.0686	−0.1364	−0.1367	−0.1990
E_3_* × 10^12^/TPa	−0.1235	−0.2410	−0.2655	−0.3740
*V_1_*	0.2517	0.4019	0.5325	0.6465
*V_2_*	1.2982	2.4937	2.8220	3.9513
*V_3_*	−0.9521	−1.7533	−2.0136	−2.7627
P_1_* × 10^3^/TPa	−1.8113	−3.5541	−3.9105	−5.5364
P_2_* × 10^3^/TPa	−0.0761	−0.1515	−0.1603	−0.2299
P_3_* × 10^3^/TPa	−0.0949	−0.1785	−0.2010	−0.2764
*H*/nm	4.5777	9.8330	10.5190	15.5773
*RR*	−1.1605	−2.6376	−2.6055	−4.1091
T_1_* × 10^−2^/nm	1.4620	2.7964	3.0736	4.2942

**Table 4:** Statistic results of Monte Carlo simulation in the self-adaptive umbrella model.

	*F_1_*/THz	*F_2_*/THz	*F_3_*/THz	*F_4_*/THz
Mean	4.8615	9.5554	10.5010	14.8827
Minimum	0.1034	0.2004	0.2233	0.3137
Maximum	31.0352	60.3568	66.4297	93.6672
Variance	3.7665	7.3630	8.0991	11.4460
Gupta [38]	1.7581	4.0706	4.7201	7.0325
Khatibi [39]	1.6030	2.4970	2.5980	3.5770
Liu [40]	1.6081	3.7232	4.3172	6.4323
Kudin [41]	1.5818	3.6623	4.2466	6.3271
Wei [37]	1.5946	3.6921	4.2811	6.3786
Cadelano [42]	1.5649	3.6232	4.2012	6.2595
Reddy [18]	1.3869	3.2111	3.7234	5.5475
Chu [43]	1.7282	3.2925	3.7442	5.1892
